# Supplementary material for: Symbiotic fouling of Vetulicola, an early Cambrian nektonic animal
Source: Commun Biol. 2020 Sep 18;3:517. doi: 10.1038/s42003-020-01244-1 (PMC7501249; doi:10.1038/s42003-020-01244-1)
Supplement: Supplementary file 1 — Supplementary Information [file 42003_2020_1244_MOESM1_ESM.docx]

**Symbiotic fouling of *Vetulicola*, an early Cambrian nektonic animal**

**Supplementary figures**

**
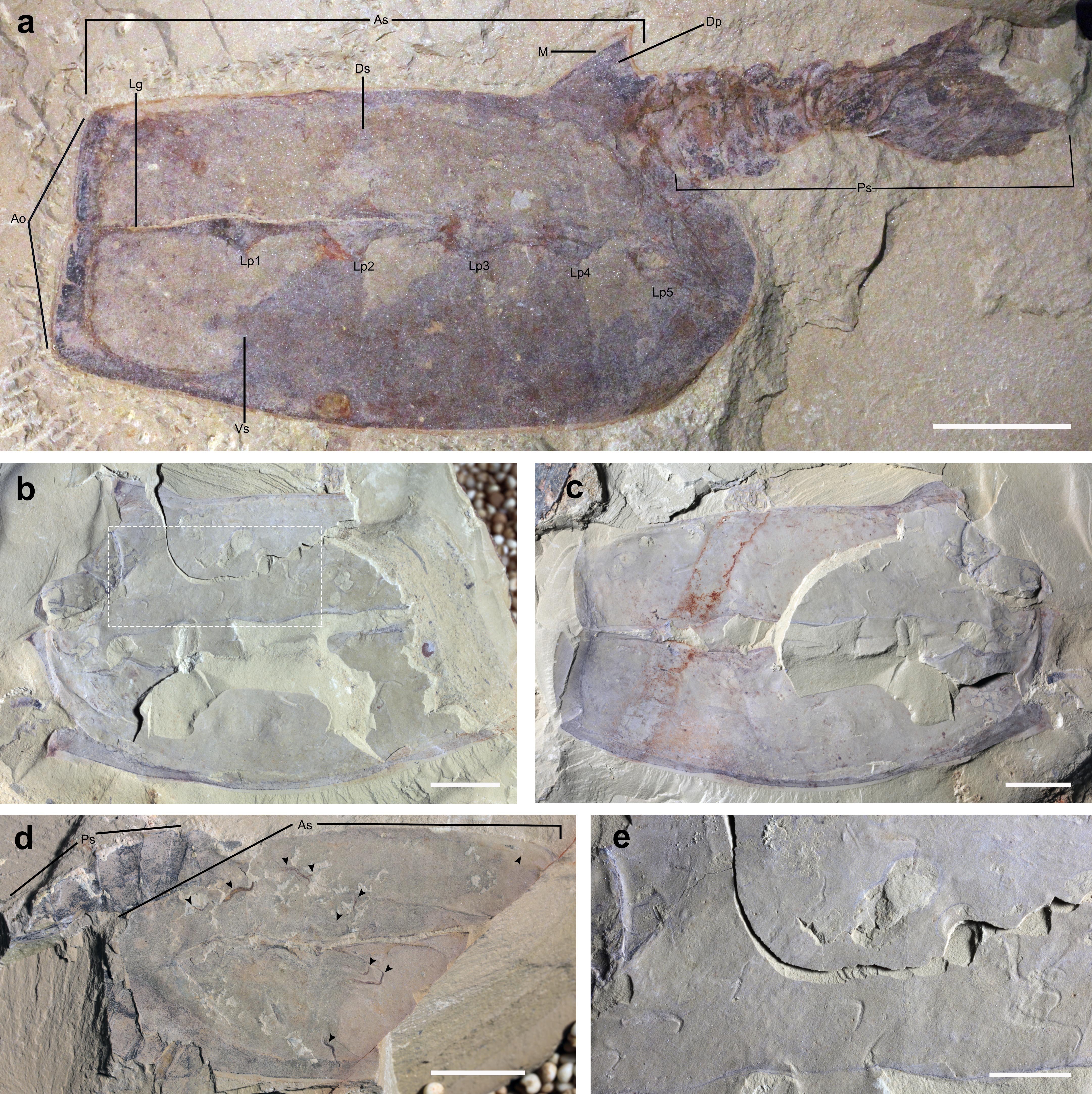
**

**Supplementary Figure 1 | *Vetulicola rectangulata* infested by *Vermilituus* *gregarius*. a, YKLP 13254(b)**, overall morphology**. b,e, YKLP 10906(a),** interior surface of the left side (dorsal to top) of the anterior section, and close-up of the orientation of J-shaped *V. gregarius* tubes (here only preserved as moulds), showing the predominant orientation of these towards the posterior. **c, YKLP 10906(b)**, interior surface of the right side of the anterior section and partial sediment fill. **d, YKLP 13076,** distribution of 10 *V. gregarius* (arrowed) in the anterior section. Abbreviations are: Ao - anterior opening, As - anterior section, Dp - posterodorsal projection, Ds - dorsal section, Lg - lateral groove, Lp - lateral pouch, M - membrane, Ps - posterior section, Vs - ventral section. Scale bars are: a-d, 1 cm; e, 5 mm.

**
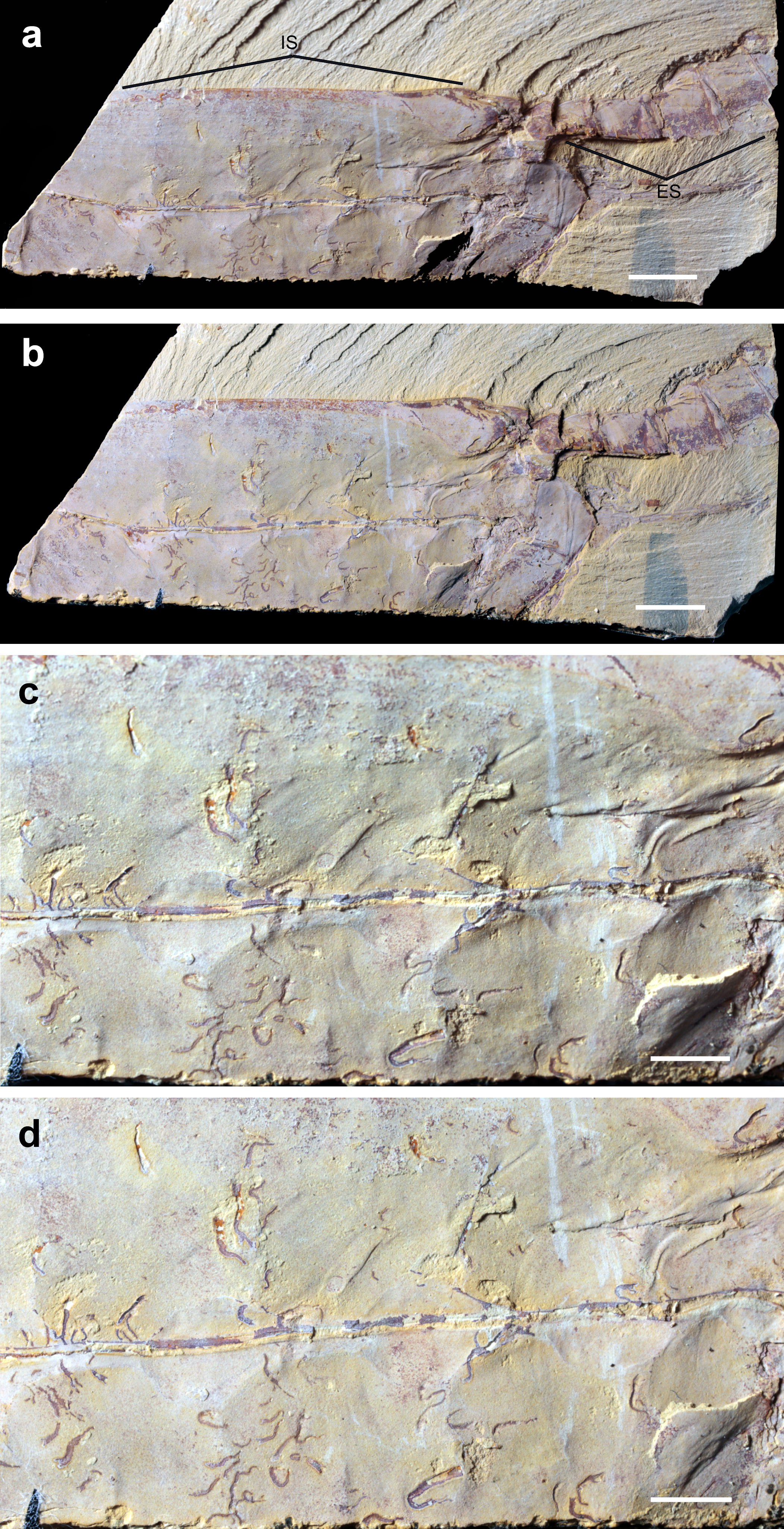
**

**Supplementary Figure** **2 | *Vetulicola rectangulata* YKLP 13075 showing taphonomic relationships with *Vermilituus gregarius*.** Stereo images have a tilt of 20 degrees to emphasise that both the worms and the *Vetulicola* are 3-dimensional. **a,b,** lateral view of whole specimen (stereo-pair) and **c,d,** close-up of anterior section (stereo-pair) respectively. This is a composite mould with external (ES) and internal surfaces (IS) preserved. Scale bars are: a,b, 1cm; c,d, 5mm.

**
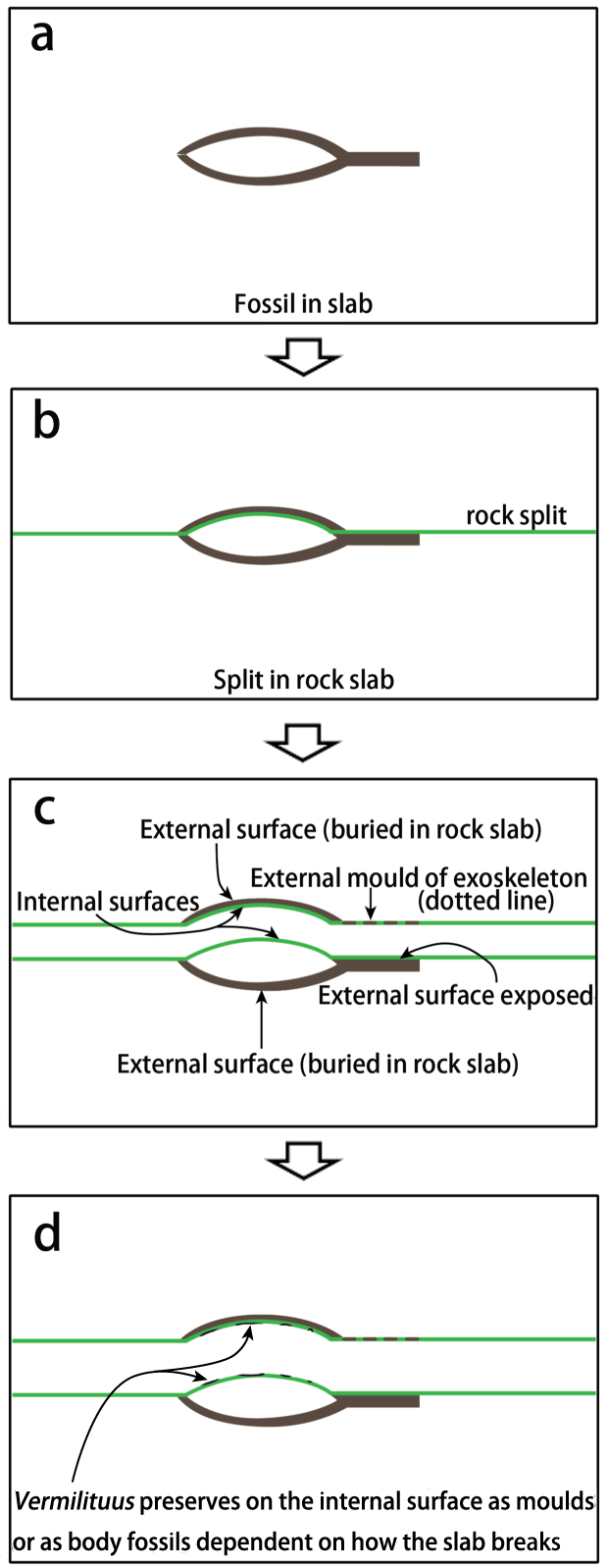
**

**Supplementary Figure** **3 | Style of preservation of *Vetulicola* in the Chengjiang biota. a,** Fossil *Vetulicola* entombed in rock slab. **b,c,** Rock splits through the fossil, so that internal and external surfaces are present in both slabs. **d,** *Vermilituus gregarius* is preserved on the internal surfaces of the fossils.

**
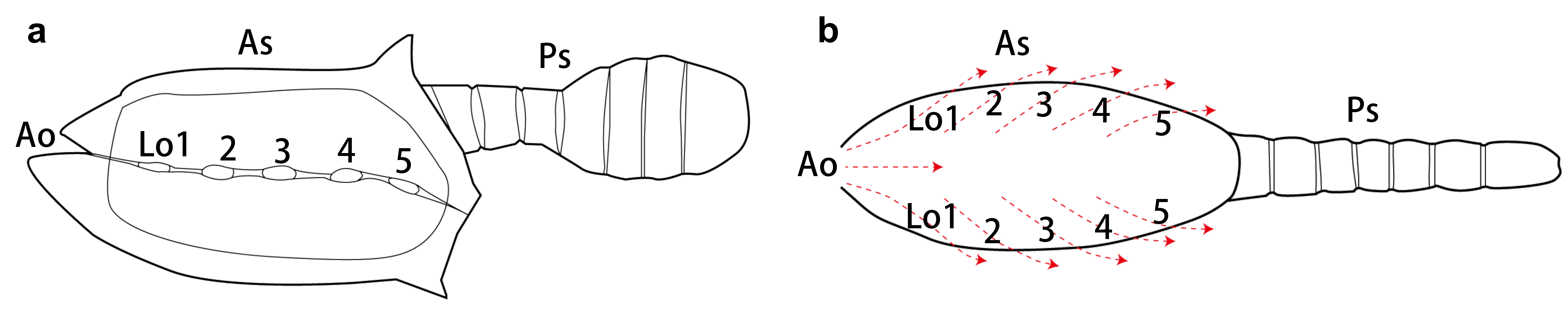
Supplementary Figure** **4 | Reconstructions of *Vetulicola cuneata*. a,** lateral view. **b,** Schematic horizontal section through the lateral midline of the anterior section with inferred water flow after^1^, but not showing the internal soft anatomy of that reconstruction. Abbreviations are: Ao: anterior opening; Lo1-5: lateral openings 1-5; As: anterior section; Ps, posterior section.

**Supplementary notes**

**Systematic affinities of *Vermilituus* *gregarius***

The systematic relationships of *Vermilituus gregarius* are uncertain. Aggregating and tubular organisms that likely represent animals are present in the terminal Neoproterozoic^2^, but none of these have comparable morphology to *V. gregarius*. Thus, *Sinotubulites* forms a cylindrical tube that is open at both ends and has an outer and inner wall structure^3^. *Wutubus* is also essentially cylindrical, with a narrower conical apex, and a truncated adapical end^4^. *Conotubus* is superficially similar to *V. gregarius*, in that it produces centimetre-sized conical tubes^5^, but these are formed from a series of nested cylindrical to funnel-shaped tube walls unlike *V. gregarius*, but reminiscent of *Cloudina*. *Corumbella* is tetraradial with longitudinal groves extending the length of the tube^6^.

By contrast to Neoproterozic tubular organisms, several groups of Phanerozoic organisms resemble *V. gregarius* in producing vermiform tubular skeletons with an annulated (growth increment) structure, including annelid worms^7^, microconchids^8^, cornulitids^9^, hederelloids^10^, trypanoporids^11^ and foraminifera^12^. Based on the style of aggregation, the type of coiling, attachment to the substrate, and slow expansion rate, *V. gregarius* superficially resembles the tubular structures of serpulid and sabellid polychaete annelids, though the earliest confirmed record of the former is Middle Triassic, and of the latter is Late Carboniferous^7^. The strongly 3-D morphology of *V. gregarius* tubes (Fig. 1c,e,f,k) suggests they may have been biomineralized, though no trace of shell material has been detected. SEM analysis reveals no evidence of wall microstructure, of internal structure, or whether the tube is open at the narrow end (Fig. 1g,i-k), and this combined with the age of the material means that comparison with serpulid annelids is speculative (see also^8,13^). Annelid worms are rare in the Chengjiang biota. Previously, six taxa have been formally described, but none of these are tube-bearing^14,15,16^. The Chengjiang priapulid *Paraselkirkia* *sinica* constructs straight tubes about 1 cm long, whilst the enigmatic Chengjiang *Archotuba elongata* is 5 cm long. Neither of these are similar to *V.* *gregarius*.

*Vermilituus gregarius* shows similarity to the annulated tubular skeletons of some organisms that are thought to be lophophorates^17^, especially cornulitids, which are known from the Middle Ordovician to Late Carboniferous, and which form endosymbiotic relationships with corals^13^. The tubes of *V. gregarius* are unlike those of encrusting microconchids (Upper Ordovician to Jurassic), which generally have planispirally coiled tubes that uncoil in some species and have a bulb-like and closed origin^8,18,19,^. *V. gregarius* differs from that group and from the Devonian trypanoporids^11^ by the absence of any internal septation. Neither does *V. gregarius* resemble the branching style of the Silurian to Permian tubular hederelloids, which are thought to have an affinity with phoronid worms^10^. *Vermilituus gregarius* does not resemble the calcium phosphatic, branching, elongate and sinuous tubes of *Sphenothallus*, a possible cnidarian taxon known from the Cambrian to Permian, which may possess a basal holdfast or flattened disk, or may taper proximally to a sharp point^20^. There are morphological similarities with some of the recently described ‘J’-shaped and ‘sinusoidal’ tubular fossils from the terminal Neoproterozoic of South China^21^, though these are not found encrusting other animals. *Vermilituus gregarius* is unlike the elongate, tapering, biomineralized tubes of the organisms encrusting the external surfaces of brachiopods in the early Cambrian Guanshan Konservat-Lagerstätte of South China^22^. Therefore, at present, the worm-like animal infesting *Vetulicola* is of uncertain affinity.

**Supplemental references**

1. Ou, Q., Conway Morris, S., Han, J., Zhang, Z.F., Liu, J.N., Chen, A.L., Zhang, X.L., Shu, D.G. Evidence for gill slits and a pharynx in Cambrian vetulicolians: Implications for the early evolution of deuterostomes. *BMC Biology*, **10**, 81 (2012).

2. Wood, R., Curtis, A. Extensive metazoan reefs from the Ediacaran Nama Group, Namibia: the rise of benthic suspension feeding. *Geobiology* DOI: 10.1111/gbi.12122 (2014).

3. Cai, Y., Xiao, S., Hua, H., Yuan, X. New material of the biomineralizing tubular fossil *Sinotubulites* from the late Ediacaran Dengying Formation, South China. *Precambrian Research* **261**, 12-24 (2015).

4. Chen, Z., Zhou, C., Xiao, S., Wang, W., Guan, C., Hua, H., Yuan, X. New Ediacara fossils preserved in marine limestone and their ecological implications. *Scientific Reports* **4**, 4180 | DOI: 10.1038/srep04180 (2014).

5. Cai Y., Schiffbauer, J.D., Hua, H., Xiao, S. Morphology and paleoecology of the late Ediacaran tubular fossil *Conotubus hemiannulatus* from the Gaojiashan Lagerstätte of southern Shaanxi Province, South China. *Precambrian Research* **191**, 46**–**57 (2011).

6. Babcock, L.E., Grunow, A.M., Sadowski, G.R., Leslie, S.A. *Corumbella*, an Ediacaran-grade organism from the Late Neoproterozoic of Brazil. *Palaeogeography, Palaeoclimatology, Palaeoecology* **220**, 7-18 (2005).

7. Ippolitov, A.P., Vinn, O., Kupriyanova, E.K. Written in stone: history of serpulid polychaetes through time. *Memoirs of the Museum Victoria* **71**, 123-159 (2014).

8. Taylor, P.D., Vinn, O. Convergent morphology in small spiral worm tubes (‘*Spirorbis*’) and its palaeoenvironmental implications. *Journal of the Geological Society, London* **163**, 225–228 (2006).

9. Vinn, O. Cornulitid tubeworms from the Ordovician of eastern Baltic. *Carnets de Géologie* **Letter 2013/03**, 131-138 (2013).

10. Taylor, P., Wooster, M. Morphology and affinities of hederelloid “bryozoans”. pp. 301-309. *Virginia Museum of Natural History, Special Publication Number 15*. Martinsville, Virginia (2008).

11. Weedon, M.J. Microstructure and affinity of the enimgmatic Devonian tubular fossil *Trypanopora*. *Lethaia* **24**, 227-234 (1991).

12. Bromley, R.G. Predation and symbiosis in some Cretaceous clionid sponges. *Bulletin of the geological Society of Denmark* **19**, 398-405 (1970).

13. Vinn, O., Mõtus, M.-A. The earliest endosymbiotic mineralized tubeworms from the Silurian of Podolia, Ukraine. *Journal of Paleontology*, 82, 409-414 (2008).

14. Huang Di‐ying, Chen Jun‐yuan, Vannier, J., Saiz Salinas, J.I. Early Cambrian sipunculan worms from southwest China. *Proceedings of the Royal Society* **B271**, 1671–1676 (2004).

15. Chen J. The Dawn of Animal World. Jiangsu Science and Technology Press, Nanjing, Jiangsu Province, China (2004).

16. Han, J., Conway Morris, S., Hoyal Cuthill, J.F., Shu, D. Sclerite-bearing annelids from the lower Cambrian of South China. *Scientific Reports* **9**, 4955 (2019).

17. Vinn, O., Mutvei, H. Calcareous tube worms of the Phanerozoic. *Estonian Journal of Earth Sciences* **58**, 286-296 (2009).

18. Zaton, M., Krawczynski, W. Microconchid tubeworms across the upper Frasnian – lower Famennian interval in the Central Devonian Field, Russia. *Palaeontology* **54**, 1455-1473 (2011).

19. Zaton, M., Vinn, O., Toom, U. A new microconchid species from the Silurian of Baltica. *Estonian Journal of Earth Sciences* **65**, 115-123 (2016).

20. Stewart, S.E., Clarkson, E.N.K., Ahlgren, J., Ahlberg, P., Schoenemann, B. *Sphenothallus* from the Furongian (Cambrian) of Scandinavia, *GFF* **137**, 20-24 (2015).

21. Cai, Y.-P., Xiao, S.-H., Li, G.-X. and Hua, H. Diverse biomineralizing animals in the terminal Ediacaran Period herald the Cambrian explosion. *Geology* 47, 380-384 (2019).

22. Zhang, Z.-F., Strotz, L.C., Topper, T.P., Chen, F.-Y., Chen, Y.-L., Liang, Y., Zhang, Z.-L., Skovsted, C.B., Brock, G.A. An encrusting kleptoparasite-host interaction from the early Cambrian. Nature Communications, <https://doi.org/10.1038/s41467-020-16332-3> (2020).
