# Supplementary material for: Symbiotic fouling of Vetulicola, an early Cambrian nektonic animal
Source: Commun Biol. 2020 Sep 18;3:517. doi: 10.1038/s42003-020-01244-1 (PMC7501249; doi:10.1038/s42003-020-01244-1)
Supplement: Supplementary file 3 — Description of Additional Supplementary Files [file 42003_2020_1244_MOESM3_ESM.docx]

**Description of Additional Supplementary Files**

**File Name: Supplementary Data 1**

**Description:** Summary of host infestation of *Vetulicola rectangulata* and *V. cuneata*.
