# Supplementary material for: Symbiotic fouling of Vetulicola, an early Cambrian nektonic animal
Source: Commun Biol. 2020 Sep 18;3:517. doi: 10.1038/s42003-020-01244-1 (PMC7501249; doi:10.1038/s42003-020-01244-1)
Supplement: Supplementary file 5 — Reporting Summary [file 42003_2020_1244_MOESM5_ESM.pdf]

nature research

Corresponding author(s): Yujing Li; Peiyun Cong

Last updated by author(s): Jul 20, 2019

Reporting Summary

Nature Research wishes to improve the reproducibility of the work that we publish. This form provides structure for consistency and transparency in reporting. For further information on Nature Research policies, see Authors & Referees and the Editorial Policy Checklist.

Statistics

For all statistical analyses, confirm that the following items are present in the figure legend, table legend, main text, or Methods section.

n/a	Confirmed	
☒	☐	The exact sample size (*n*) for each experimental group/condition, given as a discrete number and unit of measurement
☒	☐	A statement on whether measurements were taken from distinct samples or whether the same sample was measured repeatedly
☒	☐	The statistical test(s) used AND whether they are one- or two-sided<br>*Only common tests should be described solely by name; describe more complex techniques in the Methods section.*
☒	☐	A description of all covariates tested
☒	☐	A description of any assumptions or corrections, such as tests of normality and adjustment for multiple comparisons
☒	☐	A full description of the statistical parameters including central tendency (e.g. means) or other basic estimates (e.g. regression coefficient) AND variation (e.g. standard deviation) or associated estimates of uncertainty (e.g. confidence intervals)
☒	☐	For null hypothesis testing, the test statistic (e.g. *F*, *t*, *r*) with confidence intervals, effect sizes, degrees of freedom and *P* value noted<br>*Give P values as exact values whenever suitable.*
☒	☐	For Bayesian analysis, information on the choice of priors and Markov chain Monte Carlo settings
☒	☐	For hierarchical and complex designs, identification of the appropriate level for tests and full reporting of outcomes
☒	☐	Estimates of effect sizes (e.g. Cohen's *d*, Pearson's *r*), indicating how they were calculated

Our web collection on statistics for biologists contains articles on many of the points above.

Software and code

Policy information about availability of computer code

Data collection	not applicable, no code used
Data analysis	not applicable, no code used

For manuscripts utilizing custom algorithms or software that are central to the research but not yet described in published literature, software must be made available to editors/reviewers. We strongly encourage code deposition in a community repository (e.g. GitHub). See the Nature Research guidelines for submitting code & software for further information.

Data

Policy information about availability of data

All manuscripts must include a data availability statement. This statement should provide the following information, where applicable:

- Accession codes, unique identifiers, or web links for publicly available datasets
- A list of figures that have associated raw data
- A description of any restrictions on data availability

Specimens YKLP 13073-13077, YKLP 13079, YKLP 13082-13089, YKLP 13254 and YKLP 10906 are deposited at the Yunnan Key Laboratory for Palaeobiology, Yunnan University, Kunming, China. Specimens CJHMD 00031-00034 are housed at the Chengjiang Fossil Museum of the Management Committee of the Chengjiang World Heritage Fossil Site, China

Field-specific reporting

Please select the one below that is the best fit for your research. If you are not sure, read the appropriate sections before making your selection.

☐ Life sciences ☐ Behavioural & social sciences ☒ Ecological, evolutionary & environmental sciences

For a reference copy of the document with all sections, see nature.com/documents/nr-reporting-summary-flat.pdf

Ecological, evolutionary & environmental sciences study design

All studies must disclose on these points even when the disclosure is negative.

Study description	The study is based on fossil material from the Chengjiang Biota, China, as detailed in the manuscript.
Research sample	About 192 specimens from Ercaicun, 75 from Mafang, and 10 from Jianshan associated with Vetulicola rectangulata, all in the collections of the Yunnan Key Laboratory for Palaeobiology (YKLP). And 17 specimens from Xiaolantian and 55 specimens from Heimadi associated with Vetulicola cuneata, all in the collections of the Chengjiang County Museum (CJHMD, Supplementary Table 1)
Sampling strategy	Targeted collection of fossil horizons known to bear soft-bodied Chengjiang organisms.
Data collection	The fossils were collected from outcrops of the Yu'anshan Member, Chiungchussu Formation, Eoredlichia-Wutingaspis trilobite Biozone, Cambrian Series 2, Stage 3, Yunnan Province, China (Supplementary Table 1). All specimens are from 'event beds'. Some specimens were prepared mechanically with needles under a stereomicroscope.
Timing and spatial scale	Samples have been collected over several years by targeted analysis of key horizons in the Chengjiang Biota. Such fossils are exceptionally rare, so to accumulate the database presented here takes several decades of collecting.
Data exclusions	Not data exclusions
Reproducibility	All fossil materials described are available for study in the Yunnan Key Laboratory for Palaeobiology and the Chengjiang Fossil Museum
Randomization	Not applicable
Blinding	Not applicable

Did the study involve field work? ☒ Yes ☐ No

Field work, collection and transport

Field conditions	Sampling of rock successions in the Chengjiang and Kunming area, Yunnan Province, South China
Location	Chengjiang and Kunming, Yunnan Province, China
Access and import/export	All local permissions were obtained through the support of the Yunnan Key Laboratory for Palaeobiology and the Chengjiang Fossil Museum
Disturbance	Limited disturbance and full permissions obtained for sampling the rock from local land owners

Reporting for specific materials, systems and methods

We require information from authors about some types of materials, experimental systems and methods used in many studies. Here, indicate whether each material, system or method listed is relevant to your study. If you are not sure if a list item applies to your research, read the appropriate section before selecting a response.

Materials & experimental systems

n/a	Involved in the study
☒	☐ Antibodies
☒	☐ Eukaryotic cell lines
☒	☐ Palaeontology
☒	☐ Animals and other organisms
☒	☐ Human research participants
☒	☐ Clinical data

Methods

n/a	Involved in the study
☒	☐ ChIP-seq
☒	☐ Flow cytometry
☒	☐ MRI-based neuroimaging
